# Analysis of Hand and Wrist Postural Synergies in Tolerance Grasping of Various Objects

**DOI:** 10.1371/journal.pone.0161772

**Published:** 2016-08-31

**Authors:** Yuan Liu, Li Jiang, Dapeng Yang, Hong Liu

**Affiliations:** State Key Laboratory of Robotics and System, Harbin Institute of Technology, Harbin, Heilongjiang, P. R. China; Shanghai Jiao Tong University, CHINA

## Abstract

Human can successfully grasp various objects in different acceptable relative positions between human hand and objects. This grasp functionality can be described as the grasp tolerance of human hand, which is a significant functionality of human grasp. To understand the motor control of human hand completely, an analysis of hand and wrist postural synergies in tolerance grasping of various objects is needed. Ten healthy right-handed subjects were asked to perform the tolerance grasping with right hand using 6 objects of different shapes, sizes and relative positions between human hand and objects. Subjects were wearing CyberGlove attaching motion tracker on right hand, allowing a measurement of the hand and wrist postures. Correlation analysis of joints and inter-joint/inter-finger modules were carried on to explore the coordination between joints or modules. As the correlation between hand and wrist module is not obvious in tolerance grasping, individual analysis of wrist synergies would be more practical. In this case, postural synergies of hand and wrist were then presented separately through principal component analysis (PCA), expressed through the principal component (PC) information transmitted ratio, PC elements distribution and reconstructed angle error of joints. Results on correlation comparison of different module movements can be well explained by the influence factors of the joint movement correlation. Moreover, correlation analysis of joints and modules showed the wrist module had the lowest correlation among all inter-finger and inter-joint modules. Hand and wrist postures were both sufficient to be described by a few principal components. In terms of the PC elements distribution of hand postures, compared with previous investigations, there was a greater proportion of movement in the thumb joints especially the interphalangeal (IP) and opposition rotation (ROT) joint. The research could serve to a complete understanding of hand grasp, and the design, control of the anthropomorphic hand and wrist.

## Introduction

In daily life, because of the arbitrary reaching [1–3], task requirement and space-constraint grasp, the objects are grasped in different relative positions between human hand and objects. Even sometimes the relative distance is large, people still can accomplish the grasp as long as it is within the acceptable range (termed as grasp tolerance, hereafter). As a part of grasp functionality, the grasp tolerance is necessary to be considered. In order to further verify the necessity, we had a comparison of the grasp postures between the two statuses in grasp tolerance and in perfect status (grasp the objects in a self-thinking perfect posture without considering the tolerance) in our previous research [4]. We found that, only when the relative position is fully considered, the specific hand postures with acceptable grasp tolerance could be faithfully reconstructed [4]. Therefore, the grasp tolerance is necessary to be considered as a significant functionality of human grasp.

For exploring the functionality of human grasp more completely, lots of investigations have been presented. Human grasp functionality is primarily related to the applications such as biomechanics, hand surgery, and rehabilitation [5]. In these studies, hand grasp functionality is mainly reflected on two aspects: grasp adaptability to the objects of different shapes, sizes, and control principles [6–12]. The grasp adaptability means people can adopt different grips to adjust objects of different shapes and sizes. While to the different tasks of the same object, people may adopt different grips to adjust to the changing force/torque conditions. In this case, the control principles: stability and dexterity, are proposed [7]. In practice, the control principles are reflected on the contact areas between human hand and objects. According to the different contact areas on a hand, the grasps can be mainly divided into three categories: power grip category, intermediate grip category and precision grip category [8]. For power grip category, the contact area contains a larger area including a part of the palm compared with the precision grip category. For intermediate grip category, it is mainly reflected on a finger-side contact [8]. Based on the previous extensive studies, Feix et al. had a more comprehensive review and proposed a comprehensive taxonomy to represent the human grasp functionality [10]. However, the grasp tolerance is not considered in the taxonomy. In neuroscience, even if the influence of extrinsic object properties (relative position) has been paid attention [13] and some achievements have been already obtained [14]. The relative position merely means the position between a human body and an object instead of that between human hand and object, and was utilized to investigate the influence to a reach-to-grasp action [14] instead of exploring tolerance grasping as a functionality of human hand. Therefore, although there have been extensive studies on grasp functionality, the grasp tolerance has seldom been investigated as a part of grasp functionality. In addition, considering the grasp tolerance, different relative positions will make the contact area different (as aforementioned large, small or finger-side contact), which embody the control principles as a parenthesis. In this case, the grasp postures collected from the tolerance grasping of various objects could represent the grasp functionality of human hand more completely since the grasp tolerance, control principles and grasp adaptability (to different object sizes and shapes) are all considered.

As opposed to the previous researches of preselected objects grasping, Zheng et al. [15], Bullock et al. [5] [16] and Feix et al. [1] [17] utilized the camera to record totally 27.7 hours grasp postures of two housekeepers and two machinists during a wide range of unstructured tasks. Feix et al. proposed that the previous sub-categorization of grasps into power, intermediate, and precision categories may not be appropriate. The research shows that while power grasps are frequently used for heavy objects, sometimes they can still be quite practical for small and light weight objects. The grasps are generally more multi-functional than that in traditional minds [17]. The more multi-functionalities of grasps also might be partly explained by the existence of grasp tolerance in our daily grasping. Considering the grasp tolerance, due to the arbitrary reaching, task requirement or space-constraint grasp, power grasps were more suitable or even sometimes have to be used to grasp the small and light weight objects. Therefore, the grasp tolerance is also one likely complement of common sub-categorization, to represent grasp functionality more completely.

For fully exploring the human hand grasp functionality with suitable complexity, an effective analysis and evaluation tool is necessary. Since a human hand has more than 24 degree of freedoms (DoFs), synergies have become one fundamental solution to overcome the redundancy of the hand motor system. Originally, the inter-joint coordination, as a fundamental manner of human motor control, is proposed by Berstein [9]. The coordination of motor control can be explained by “synergy control” which can be included on several aspects: kinematic, force, eletromyographic (EMG) activity of hand muscles, and neurophysiology of cortical and spinal neurons [18]. Among them, the kinematic synergy is the most fundamental and critical one. Santello et al. [19] reported that hand postures could be reconstructed by a small number of principal components. The first two principal components accounted for ~84% of the variance over 57 objects grasp postures. Following the research of Santello, hand postures in reach-to-grasp action [20], angular velocities of finger joints [21] [22] [23] [24], hand postures in bimanual manipulation of various objects [25] can also be described by a small number of postural synergies. However, on previous investigations of postural synergies, the tolerance grasping of various objects is not presented.

Consequently, we have an analysis of the hand and wrist postural synergies in tolerance grasping of various objects in this paper. Firstly, we constructed a grasp database (contained hand and wrist postures) in the consideration of grasp tolerance, control principles (as a parenthesis of grasp tolerance) and grasp adaptability. Then, we performed correlation analysis among joints and modules newly defined. On the basis of correlation analysis results, the synergy schemes of hand and wrist postures were analyzed separately by the PC information transmitted ratio and PC elements distribution. Furthermore, to understand the posture reconstruction performance, the reconstructed joint angle error under the postural synergies is reported. The experimental results demonstrated that the coordination between wrist and hand joints is not obvious in tolerance grasping, the hand and wrist postures can be separately well reconstructed by first four hand principal components (PCs) and first two wrist PCs with small reconstructed angle errors (mostly within 10°) in the tolerance grasping of various objects. The research can be applied on some other areas. As the tolerance grasping of various objects can represent human grasp functionality more completely, rehabilitation practitioner can use the grasp database constructed by tolerance grasping to build a clinical assessment tool of hand, wrist pathologic and prosthetic hand performance. The designer of the anthropomorphic hand and wrist can design and control the anthropomorphic hand and wrist by the synergy schemes and correlation results.

## Materials and Methods

### Recording and reconstruction system for human hand grasp

**Fig 1:**
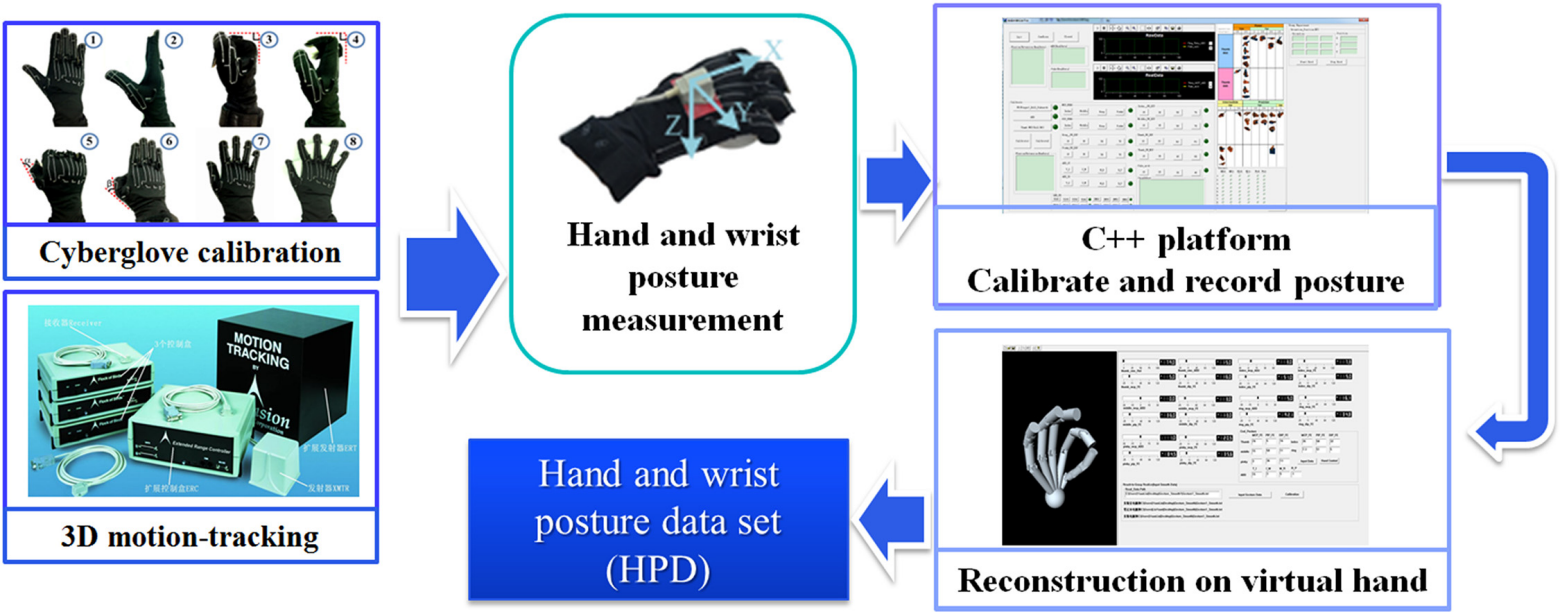
Recording and reconstruction system on human grasp.

To record the hand grasp information more conveniently and accurately, a recording and reconstruction system for human hand grasp was constructed (see Fig 1). The system consists of three parts: CyberGlove to measure the hand grasp posture, 3D motion-tracking (Fastrack Polhemus 3D motion-tracking system) to measure the wrist posture, and PC to calibrate, record and reconstruct the posture by the self-developed recording and reconstruction software. At the beginning of experiment, the subject was asked to put on the CyberGlove. Then, a calibration on CyberGlove was carried on step by step by a self-developed C++ software of calibration. Moreover, the mapping relation between sensor raw data and joint actual angle was obtained automatically by this C++ software. To guarantee the accuracy on mapping relation, a key-posture CyberGlove calibration procedure was developed on the basis of Huenerfauth [26]. The calibration procedure will be introduced in later part in detail. After finishing the CyberGlove calibration, the subject was asked to perform different kinds of grasp tasks. Simultaneously, hand and wrist postures were recorded by the C++ software. At last, to guarantee the accuracy of grasp posture, the hand and wrist postures verified by the self-developed posture reconstruction software were imported to the hand and wrist posture data set (HPD).

**Fig 2:**
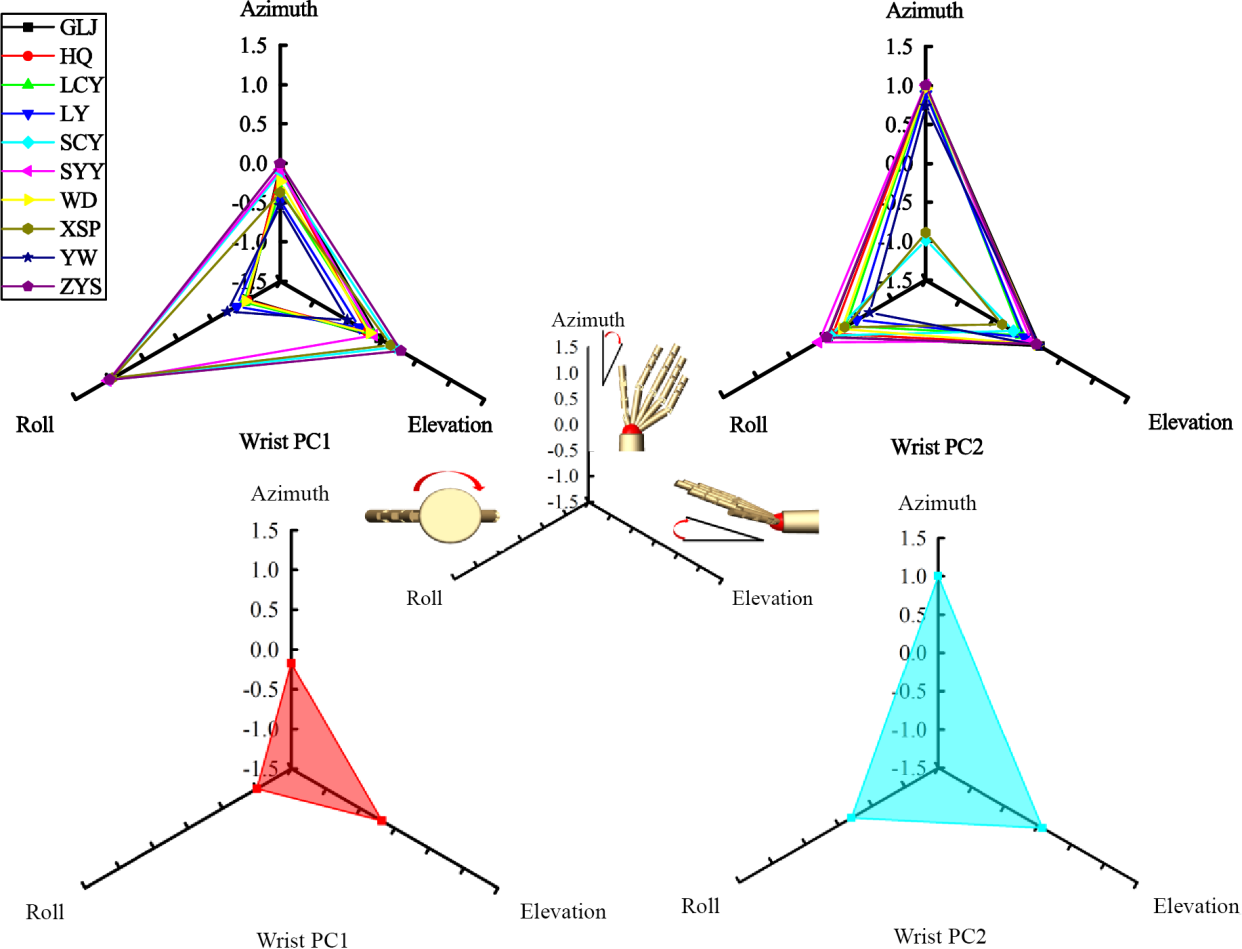
First two PCs of wrist postures to each subject and all ten subjects. The abbreviations represent the name of each subject.

Hand grasp postures contained 15 joints information were actually recorded by CyberGlove III (Virtual Technologies, Palo Alto, CA) at a resolution of <0.1° and sampled at 100 Hz each. The following joint angles were measured: flexion and extension of proximal interphalangeal (PIP) joints and metacarpo-phalangeal (MCP) joints of digit II-V, as well as the interphalangeal (IP) and MCP joints of the thumb (digit I), rotation (ROT) of thumb across the palm and abduction/adduction (ABD) between each two adjacent digits of digit I-V. Simultaneously, wrist postures (hand orientation) were recorded by the Fastrack Polhemus 3D motion-tracking system (acquisition rate: 100 Hz, positional accuracy: 0.8 mm RMS, rotational accuracy: 0.15° RMS; Roby-Brami et al. 2000). The Polhemus receiver was attached to the back of CyberGlove. Wrist postures were given by Euler angles of azimuth, elevation and roll. The corresponding relation between wrist joint rotation angles and Euler angles can be seen in the center of Fig 2. When the subject finished each grasp trail, the subject hand postures and wrist postures were recorded.

CyberGlove III (18 sensors gloves) was used to measure the 15 joint angles of thumb and finger joints. The C++ software we developed was used to record the joint sensor raw data of different key-postures step by step in calibration approach (S1 Table and S1 Fig), then the linear interpolation was used to construct the mapping relation between the joint sensor raw data and actual joint angle. Finally, we could obtain the mapping relation between sensor raw data and joint actual angle.

### Experimental protocol

Ten healthy subjects of right-handed (24~27 years old, 8 men and 2 women) volunteered to take part in the experiments. Each subject is of good health and has no history of neurological or motor disorders. The experimental protocol was approved by the “institutional review board (IRB)” of Harbin Institute of Technology, Harbin, P. R. China. Before the experiment, each participant of all ten subjects signed the informed consent. In our subject informed consent form, the purpose of the experiment, time required for each participant, and the experiment protocol were presented. After comprehending the all statements in our subject informed consent form, each participant could sign the informed consent if they agree to the voluntary participation request.

**Fig 3:**
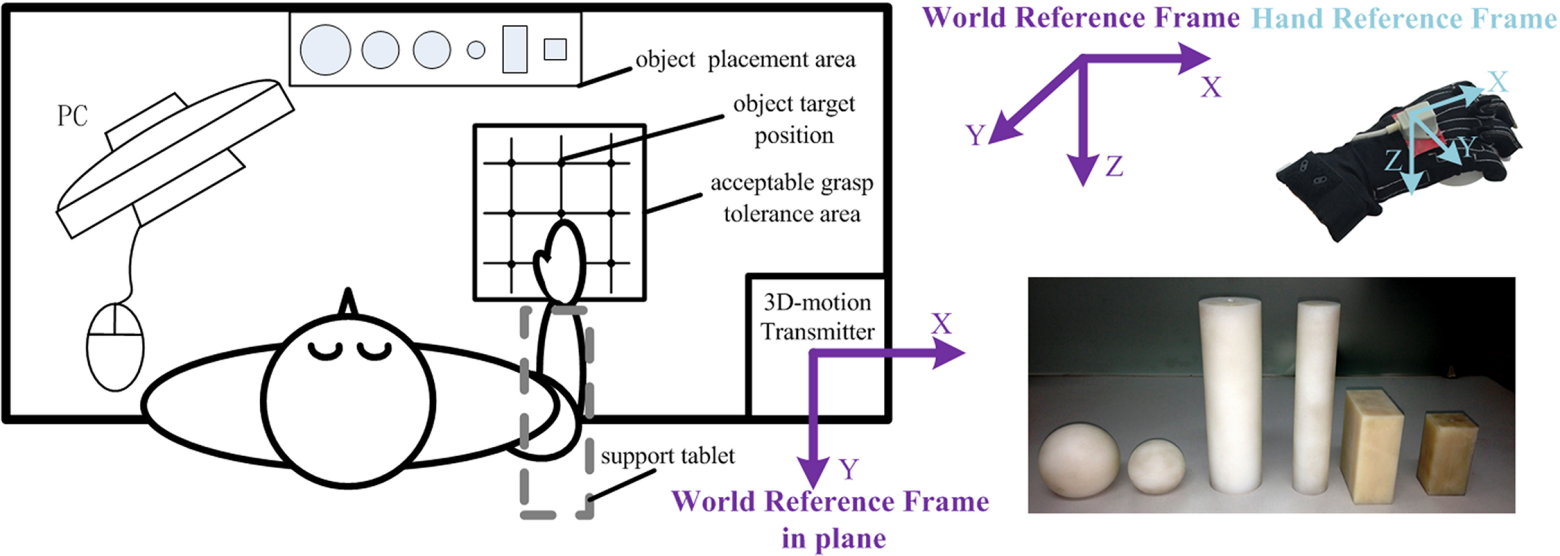
Experimental setup.

The subject sat in front of the table (Fig 3). The elbow and wrist rested on the support tablet to make the forearm horizontal, the arm was oriented in the parasagittal plane passing through the shoulder, and the hand was in a pronated position. Right wrist through the anti-static wrist strap secured to the cantilever pole of the stationary bracket through the short inelasticity rope fixed by the snap fastener in anti-static wrist strap and the bolt in the cantilever pole, which was utilized to avoid the movement of wrist. The stationary bracket consists of supporting seat, steadying bar, cantilever support tablet and pole. The whole stationary bracket is fixed to the table by the bolt compaction between the bracket supporting seat and table. The steadying bar is vertically and fixedly connected with the supporting seat. The cantilever support tablet and pole were fixed to the steadying bar and could be adjusted to different height. The distance between cantilever support tablet and pole fixing anti-static wrist strap was unchanged. The different relative heights (high, middle, low) between hand and objects were obtained by adjusting the height of cantilever support tablet and pole.

After the pre-experiments, the acceptable grasp tolerance area within which subjects can successfully grasp objects was obtained (as shown in Fig 3). The distances between the vertical lines and between the horizontal lines in the object target position area were 6cm and 4.5cm, respectively. The relative height between adjacent heights was 3cm. The grasped object target position on the plane is shown in Fig 3. The six different objects grasped in different relative positions were chosen to span the hand postures of different grasp types. The shape, size and weight of objects we chose (see Table 1) were based on the Feix et al. [1], Zheng et al. [15] and Bullock et al. [5] [16] research results to high-effectively represent the objects we grasped in daily life.

**Table 1:** Shape, size and weight of the six grasping objects.

Shape		Size (mm)	Weight (g)
**Sphere**	Large	Diameter 80	300
	Small	Diameter 60	100
**Cylinder**	Large	Diameter 60; height 200	650
	Small	Diameter 40; height 200	300
**Prism**	Large	Length:80;width:40;height:100	300
	Small	Length:40;width:40;height:100	150

Each subject was asked to grasp 6 different objects in 27 different relative positions (3x×3y×3z) between human hand and objects. The relative position between human hand and object is defined as the relative position between the center of human wrist and object center of gravity in this paper. Each object was grasped twice. In total, 324 trails (1 subjects× 6objects×27 relative distances×2repeats) across all six objects were performed by each subject over a period of ~2 h. Subjects were instructed to firstly place the object from the object placement area to the target position with their left hand, and then grasp the object with their right hands. After that, the subject was asked to hold the grasp posture (hand and wrist posture) three seconds for preliminary recording the posture. Then, subjects had to lift up the objects to ensure they could move objects successfully by the grasp posture. Once completing the verification, the hand and wrist grasp posture was finally recorded. After each grasp trail is accomplished, the subject put the object back into the original position and began the next grasp trail until all trails were accomplished. No gesture constrains were given, the grasp postures were entirely decided by subjects under the premise of stable, nature and comfortable grasping. No explicit constraints on movement velocity were given.

### Data analysis and statistics

The hand and wrist data was averaged over the two repeats across two trails. Then the human grasp posture data set in different relative positions (HPD-RP) is obtained for exploring the grasp functionality of human hand. The single one hand posture and wrist posture of one subject can be defined as *Q*_i_ = [*q*_*i*1_ *q*_*i*2_ ⋯ *q*_*in*_]^*T*^ ∈ *R*^*n*^, where n is the kinematic DoF. N is defined as the number of hand or wrist postures. For analyzing the postures totally or individually, N is equal to 1620 or 162. Therefore posture data set can be represented by *Q* = [*Q*_1_ *Q*_2_ ⋯ *Q*_*N*_]^*T*^ ∈ *R*^*N*×*n*^. All data analyses and statistics were on the base of HPD-RP.

At first, correlation analysis was utilized to assess the joint coordination of hand and wrist. To assess the correlation relation between one joint and all other joints, the absolute value of correlation coefficients between one joint and all other joints was averaged and the mean correlation coefficients of each joint (mean correlation coefficient (joint)) were obtained. Moreover, for comparing the synergies of joint muscle groups conveniently, all hand and wrist joints were divided into inter-finger modules and inter-joint modules. The inter-finger modules contain five modules: thumb module, index finger module, middle finger module, ring finger module and pinky module. Apart from thumb module, each inter-finger module contains all joints except for ABD joints of corresponding finger. The thumb module is constituted by IP, MCP, ROT and ABD joint of the thumb. The inter-joint modules contain four modules: MCP joint module, PIP joint module, ABD joint module and wrist joint module. Each inter-joint module contains all corresponding joints of digit II-V and wrist joints (given by Euler angles). Then, based on the obtained mean correlation coefficient (joint), the mean correlation coefficients of all joints in each module were averaged; finally the mean correlation coefficient (module) was obtained.

Then, principal component analyses were separately applied to hand posture (15 DoF), hand and wrist posture (18 DoF), hand posture without thumb (11 DoF). As the mean correlation coefficient (module) of wrist is low, PCA was also used to analyze the wrist posture (3 DoF) individually. The HPD-RP was analyzed by PCA from two kinds. The first kind PCA data set contained all subjects posture information which was consisted of 1620×DoF matrix (ten subjects×27 relative distance×six objects and 18, 15, 11 hand kinematic DoF and 3 wrist DoF). The second kind PCA data set contained each subject posture information which is consisted of 162×DoF matrix (one subjects×27 relative distance×six objects and 18, 15, 11 hand kinematic DoF and 3 wrist DoF) and was utilized to show the posture characteristic consistency of each subject.

In order to characterize the variance accounted by each PC, information transmitted by PCs of different number of DoF (11 DoF, 15DoF, 18 DoF and 3 DoF) were calculated.

Let C be the covariance matrix of posture data set Q:

$$C=\mathrm{cov}(Q)\in R^{n\times n}$$ (1)

Computing the largest *d* eigenvalues *δ*_*i*_ and corresponding eigenvectors *e*_*i*_ of the covariance matrix C, the hand posture and the wrist posture data set can be described by a small number of principal components:

$$\begin{array}{l} Q\approx \bar{Q}+P_{N\times d}\times E_{d\times n}\\ \;\approx \left[\begin{array}{ccc} \bar{q_{11}} & \cdots & \bar{q_{1n}}\\ \bar{q_{21}} & \cdots & \bar{q_{2n}}\\ \vdots & \cdots & \vdots \\ \vdots & \cdots & \vdots \\ \bar{q_{N1}} & \cdots & \bar{q_{Nn}} \end{array}\right]+\left[\begin{array}{cccc} s_{11} & s_{12} & \cdots & s_{1d}\\ \vdots & \vdots & \vdots & \vdots \\ s_{i1} & s_{i2} & \cdots & s_{id}\\ \vdots & \vdots & \vdots & \vdots \\ s_{N1} & s_{N2} & \cdots & s_{Nd} \end{array}\right]\left[\begin{array}{c} e_{1}^{T}\\ e_{2}^{T}\\ \vdots \\ e_{d}^{T} \end{array}\right] \end{array}$$ (2)

$${\bar{q}}_{*j}=\frac{1}{N}\sum_{i=1}^{N}q_{ij}$$ (3)

${\bar{q}}_{*j}$ is the average of joint angle of j-th kinematic DoF across N postures. *j* = 1,2,⋯,*n*. The vectors *e*_*i*_ are also called the eigenpostures in the research of postural synergies, *e*_*k*_ ∈ *R*^*n*×1^, *d* is the driving DoF of grasp posture after dimension reduction by PCA. The values *s*_*ij*_ are scalar weights which are the controlled variable on eigenposture.

The information transmitted ratio can be written as:

$$T=\sum_{i=1}^{d}\delta_{i}/\sum_{i=1}^{n}\delta_{i}$$ (4)

This metric has been presented by Eckart and Young [27]. In robotic hand design area, *T* quantified anthropomorphic motion capability of reconstruction [28].

To show the posture reconstruction performance of hand postures, the absolute reconstructed angle errors of each joint in each posture (all 1620 postures) by first four PCs of human hand joints (15DOF) were calculated. Then, in order to show the positional deviation influence on the reconstructed angle errors, the reconstructed angle errors of each joint in all 1620 postures were divided into three parts in each positional deviation orientations (X, Y or Z orientation). For example, to show the X orientations positional deviation influence on the reconstructed angle errors, the reconstructed angle errors of each joint in all 1620 postures were divided into the left, middle and right positional deviation parts. It is similar to show the positional deviation influence in Y or Z orientation. Finally, the means and standard deviations (Mean ± SD) of each joint reconstructed angle errors in each positional deviation part were calculated. To show the wrist posture reconstruction performance, similar to the aforementioned calculation method of reconstructed hand joint angle errors, only the reconstructed angle errors of each wrist joint in each posture were the reconstruction result of first two PCs of wrist joints (3 DOF).

## Results

### Correlation analysis among joint and module movements

**Fig 4:**
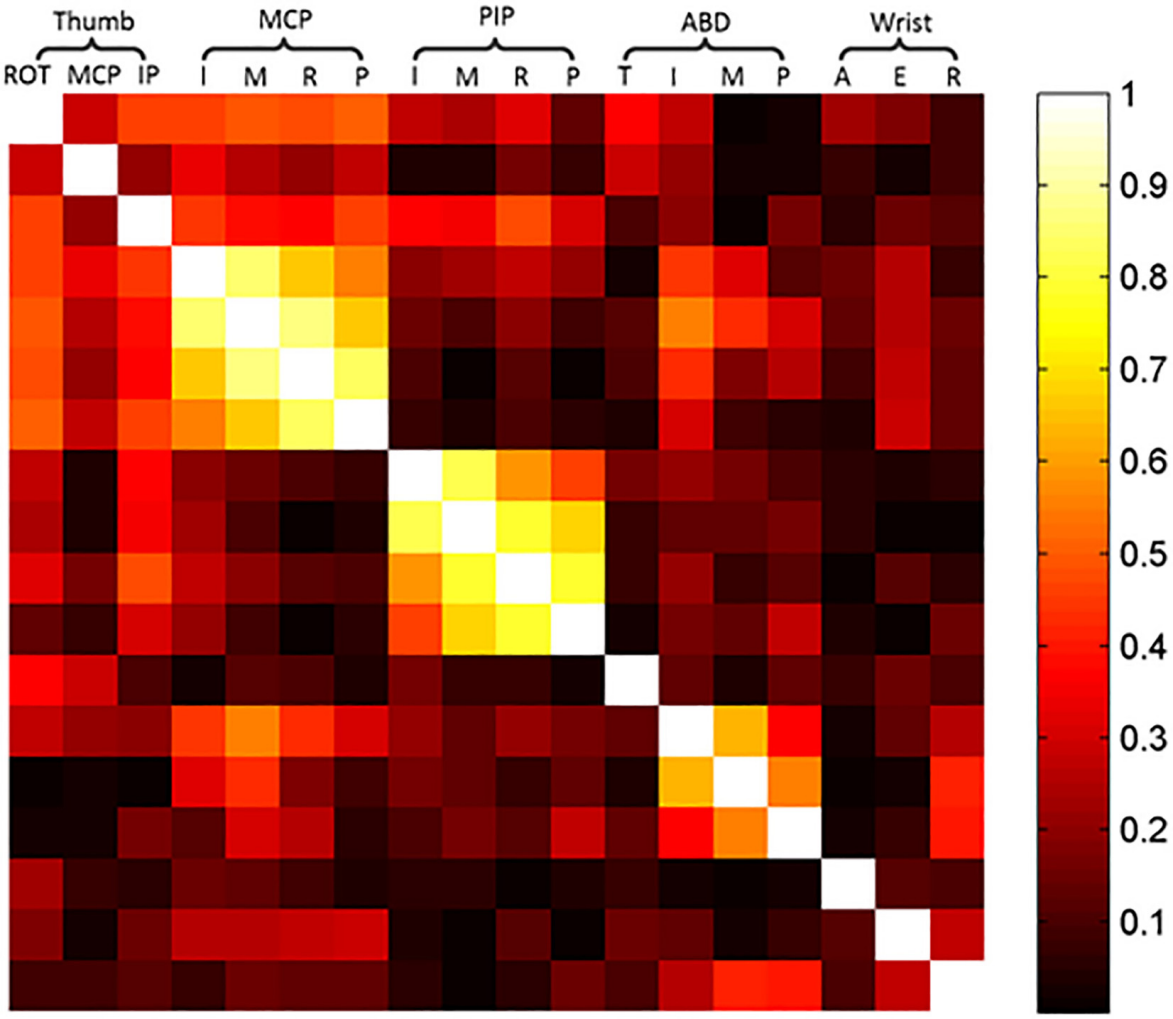
Correlation coefficients between 18 joint angles of hand and wrist. The gray scale in each square denotes the absolute coefficient of determination (r^2^) for the relation between the angles.

Fig 4 shows the correlation between 18 joint angles of hand and wrist for ten subjects. The rotation angles of MCP joints of the digit II-V were all positively correlated. Similarly, the PIP angles were positively correlated with each other, and the ABD angles between five fingers were, too. For the correlation of joints, MCP angles between adjacent fingers tended to be highly correlated (Fig 4, left upper large light color square), as were adjacent PIP angles but lower than MCP angles (Fig 4, central large light color square) and adjacent ABD angles but lower than PIP angles(Fig 4, right lower large light color square). The highest correlation coefficient among all correlation coefficients of MCP angles, PIP angles and ABD angles were 0.87 (between middle finger and ring finger), 0.81 (between index finger and middle finger) and 0.63 (between index finger and middle finger), respectively.

**Fig 5:**
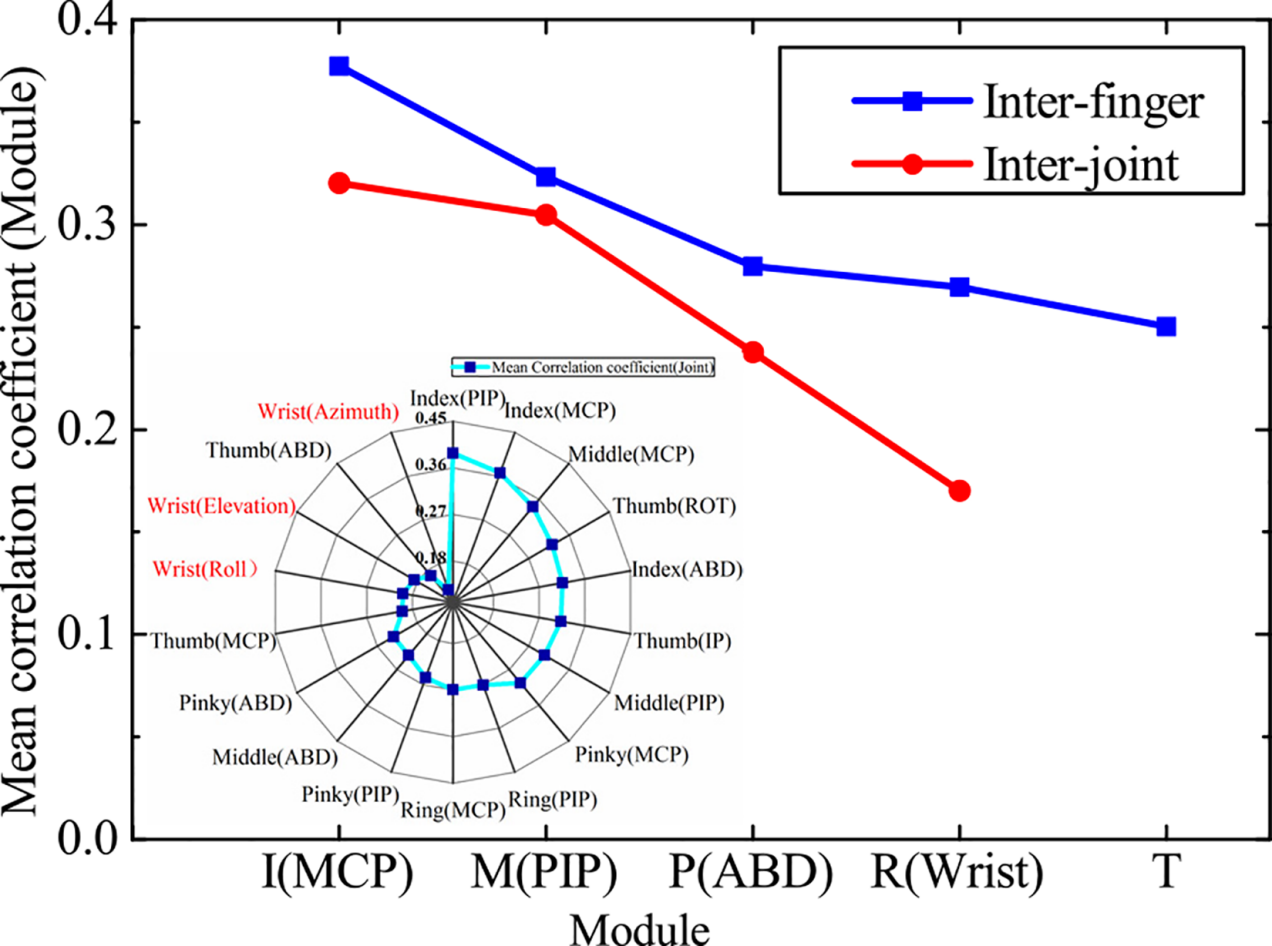
Mean correlation coefficient analysis on modules and joints. The I, M, P, R and T of abscissa denote module of inter-joint, middle figner, ring finger, pinky and thumb, respectively. The MCP, PIP, ABD and wrist denote corresponding inter-joint module.

Fig 5 illustrates the mean correlation coefficient (joint) (see left lower radar figure of the Fig 5) and the mean correlation coefficient (module) (see line-symbol figure of Fig 5). From the Fig 5, for inter-finger coordination, we can see that the module of index finger and middle finger generally had high correlation with joints of other modules, followed by modules of pinky, ring finger and thumb joints. For inter-joint coordination, the modules of MCP, PIP had a higher correlation with joints of other modules than that of ABD and wrist. In contrast, coordination correlation between wrist and hand joints was the lowest among all modules. This also can be seen from Fig 4 that the color of last three colums was grayer, which means the movement correlation between wrist and hand joints was low. This low correlation between wrist and hand joints indicated the coordination between wrist and hand was not obvious in tolerance grasping. Therefore, the hand postural synergies and wrist postural synergies were analyzed separately.

### Hand postural synergies

#### Hand posture information transmitted by PCs

**Fig 6:**
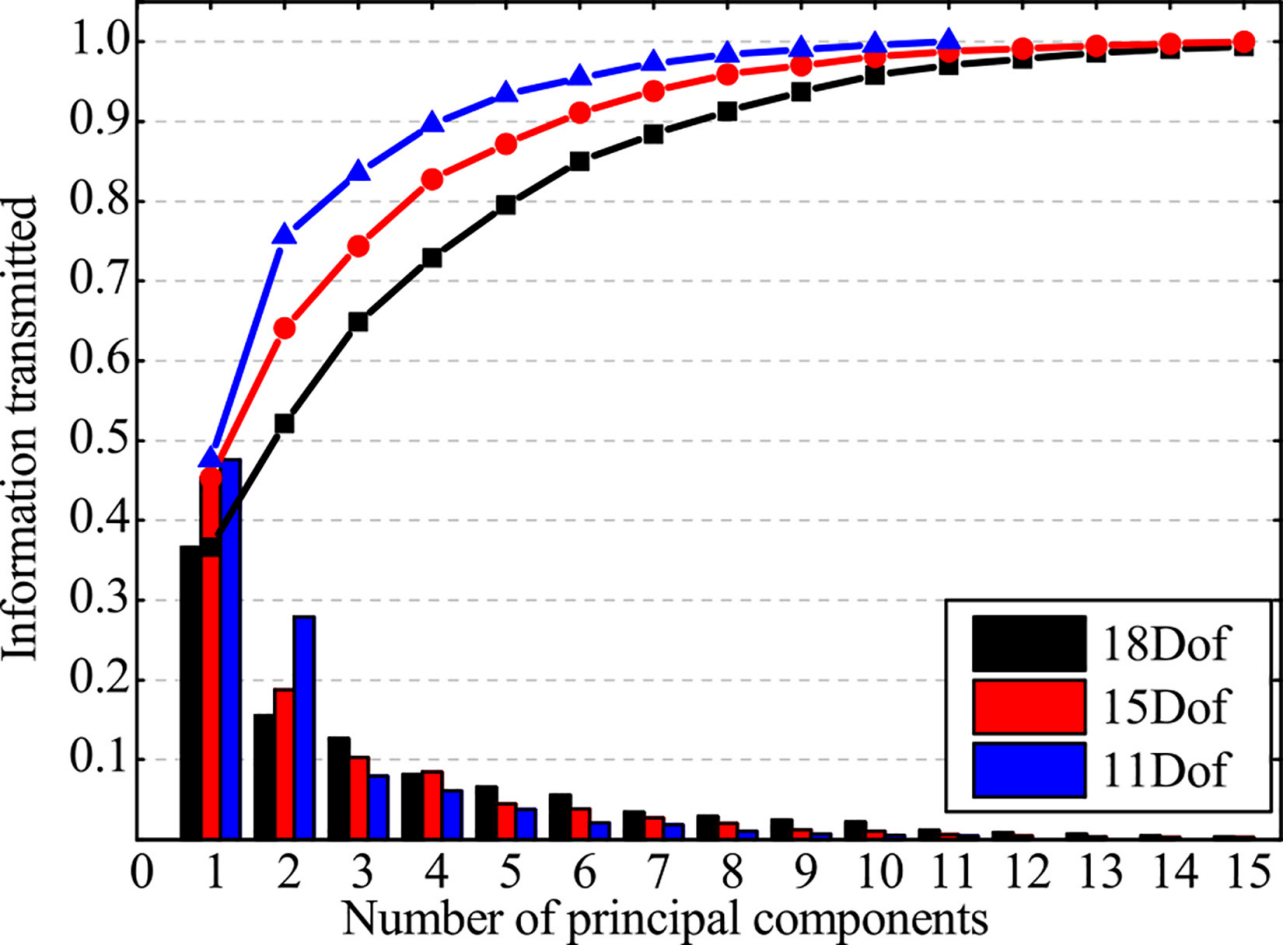
Information transmitted by each PC of postures contained different number of DOFs. The postures of different number of DOFs contained hand and wrist posture (18DoF), hand posture (15DoF), hand without thumb posture (11DoF) to all ten subjects.

The information transmitted by each PC of hand postures (15 DoF) to all ten subjects is shown in Fig 6. Principal component analysis showed a clear dimension reduction on the variable space. The first six PCs to 18 DoF, first four PCs to 15DoF, and first three PCs to 11 DoF could account for more than 80% of the postural variability. For each subject, information transmitted by PC of hand posture (15DoF) was highly consistent (S2 Fig).

#### First four PCs of hand postures

**Fig 7:**
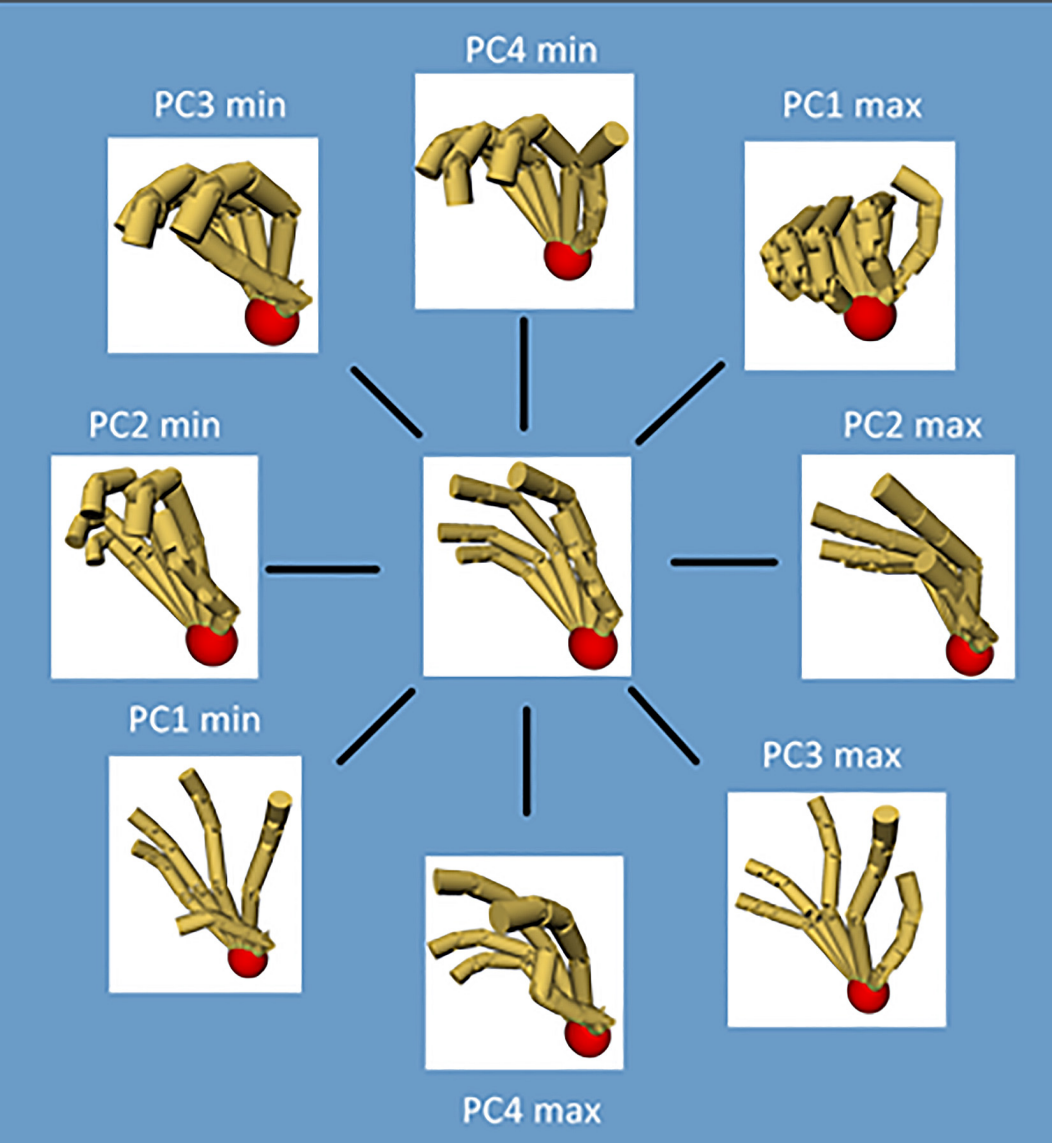
The max and min postures of the first 4 PCs on the self-developed posture reconstruction software.

Fig 7 shows the visualization of the min and max postures of the first 4 PCs of hand postures to all ten subjects (The quantified angle changing of each joint for a unit change in the amplitude of the first to fourth PC of hand postures to all ten subjects is shown in S3 Fig). The first PC was characterized by flexion of all MCP joints, PIP joints and thumb IP joint, inverse opposition motion of thumb rotation joint, and a lesser degree of adduction of index, ring and pinky. For the ADLs grasping, this could be characterized by the cylinder grasping (such as the pen, cup, handle, mug et al.) and flat thin objects grasping (such as spoon, wrench, card, dinner plate et al.). The second PC was characterized by converse move between all MCP joints and all PIP joints, this could be used to grasp flat objects (such as book, plate, brick, disk et al.) in palm-opposability posture or pad-opposability posture. The third PC was characterized by principal rotation of MCP joints of four digits and thumb rotation joint, lesser rotation of all PIP joints, abduction-adduction of index, ring and pinky. It showed an opposability grasp of sphere objects (like apple, tennis et al.). The fourth PC was mainly characterized by the abduction-adduction of index finger, ring finger, pinky and thumb, rotation of thumb IP, and opposition motion of thumb rotation joint.

#### Reconstructed angle error by first four PCs of hand postures

**Fig 8:**
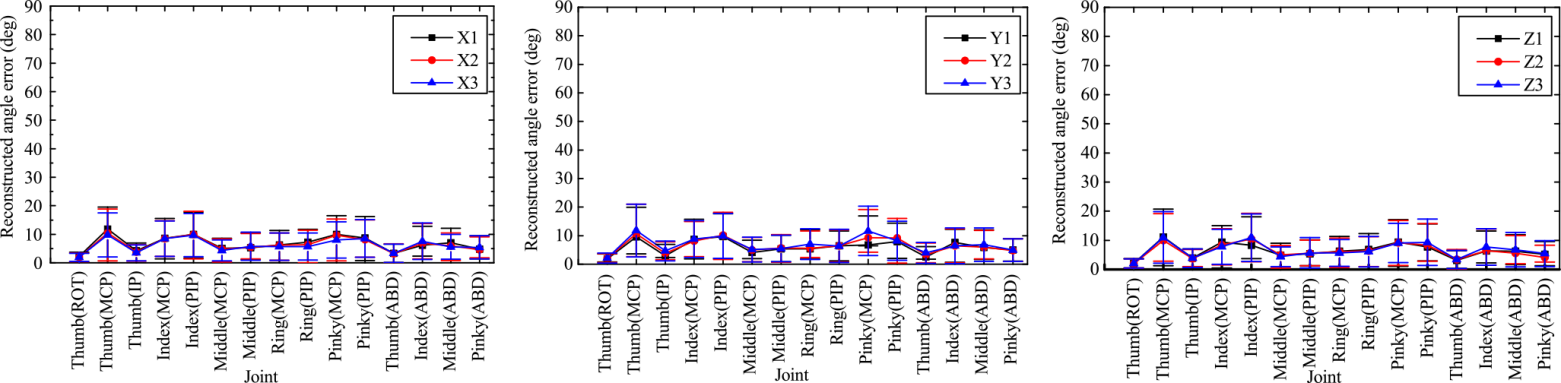
Reconstructed angle error distributions by first four PCs of hand postures (15DOF).

**Fig 9:**
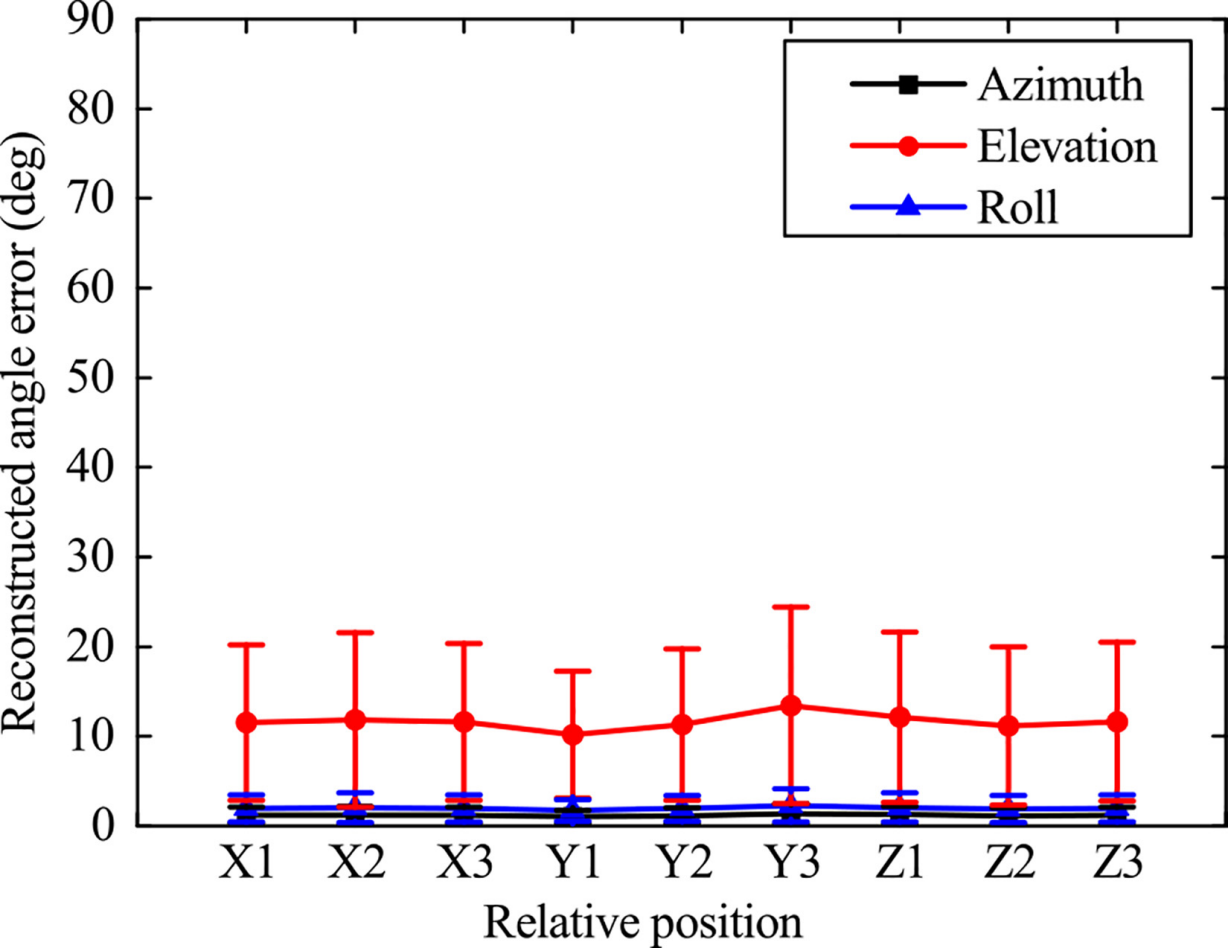
Reconstructed angle error distributions by first two PCs of wrist postures (3DOF)

The reconstructed angle error by first four PCs of hand postures (15DoF) is shown in Fig 8. The X1, X2 and X3 in Fig 8 and Fig 9 represented X positional deviation from left to right. The Y1, Y2 and Y3 in Fig 8 and Fig 9 represented Y positional deviation from distal to proximal. The Z1, Z2 and Z3 in Fig 8 and Fig 9 represented height deviation from low to high. Under the inspection of Fig 8, the distribution trends of the reconstructed angle errors in different positional deviation of X, Y and Z orientations were highly similar. The mean reconstructed angle errors of hand joint were mostly within 10°. The great reconstructed angle errors were occurred in thumb IP joint, thumb MCP joint, index PIP joint, pinky MCP joint and pinky PIP joint.

### Wrist postural synergies

#### Wrist posture information transmitted by PCs

**Fig 10:**
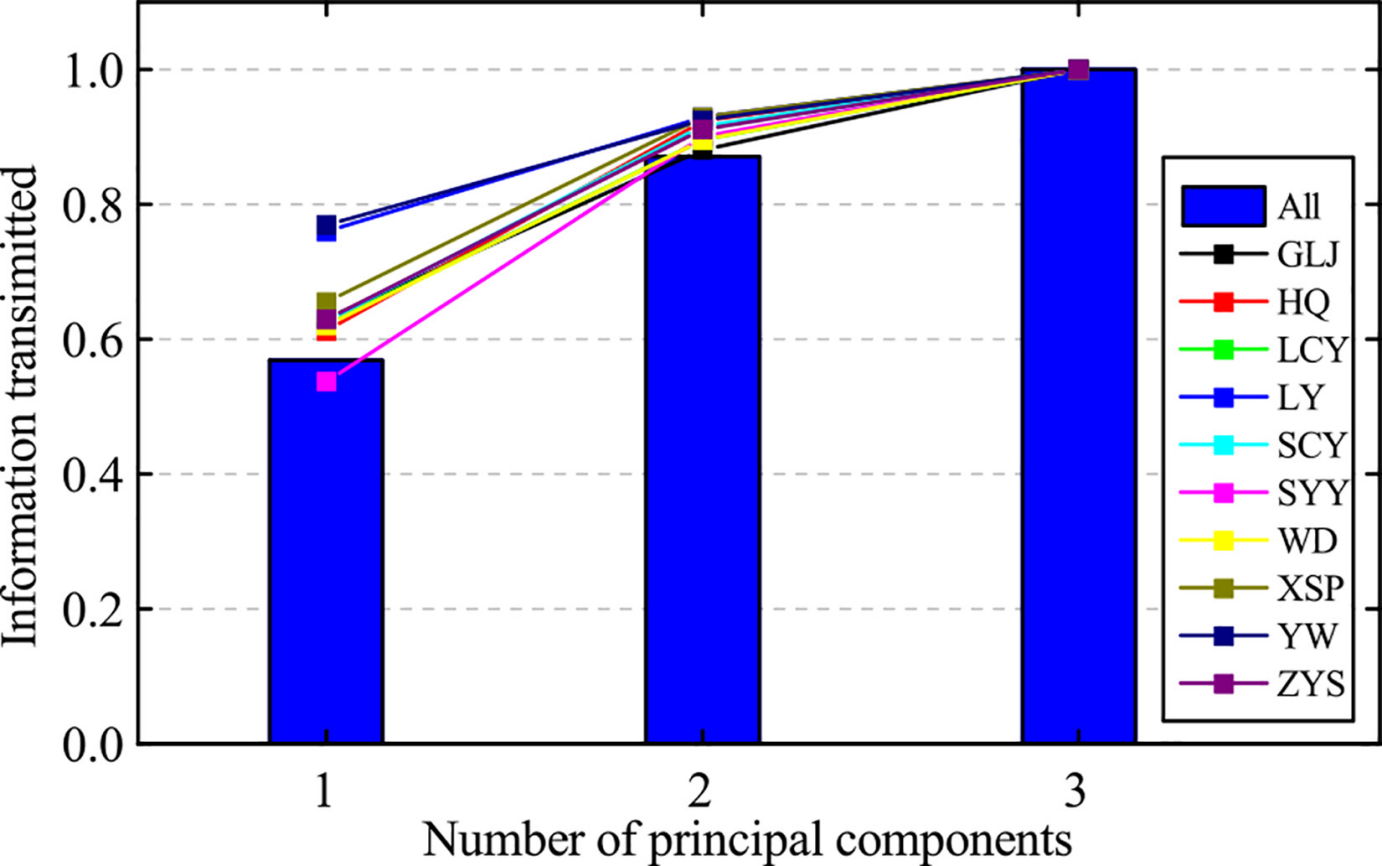
Information transmitted by PCs of wrist postures to each subject and all ten subjects. The abbreviations represent the name of each subject.

Information transmitted by PCs of wrist postures (3DoF) to each subject and all ten subjects are shown in Fig 10. The first PC of wrist postures to all ten subjects accounted for almost 60% of the variance in wrist posture. Besides, the first two PCs of wrist postures accounted for 87%. Then, Information transmitted by PCs of wrist postures of each subject is also shown on the scatter line in Fig 10.

#### First two PCs of wrist postures

First two PCs of wrist postures to each subject are shown on the top panel of Fig 2. The bottom panels of Fig 2 showed the first two PCs of wrist postures to all ten subjects. Center figure of Fig 2 showed the wrist joint rotation along the positive direction of the axis. To different subjects, wrist PCs showed lower consistence than hand PCs.

#### Reconstructed angle error by first two PCs of wrist postures

The reconstructed angle errors by first two PCs of wrist postures (3DoF) were shown in Fig 9. To different positional deviation in X, Y and Z orientations, the reconstructed angle error distribution trends of azimuth, elevation and roll were also similar, while the reconstructed angle error of elevation was much larger than azimuth and roll. The mean reconstructed angle error of elevation in different relative positions was about 10° and larger than that of azimuth and roll (less than 5°).

## Discussion

### Correlations of joint and module movements

Results on the correlations between 15 hand joints angles of ten subjects are consistent with previous study showing that MCP angles, PIP angles and ABD angles between adjacent fingers tended to be highly correlated [19].

For comparing the correlations of joints, the correlation coefficients of joints are reported in previous studies [19]. However, this is very difficult to be applied to some other applied research fields like anthropomorphic hand design because of the high-fragment and lack-contrast. In some other researches like robotic area, most of investigations are to build models of electromyogram (EMG) signals of natural movements for controlling myoelectric prostheses [29] [30] [31]. The correlations between different functional muscles are seen as a natural phenomenon and seldom investigated. In order to overcome the high-fragment and lack-contrast of previous correlation research, we grouped the joints in inter-joint and inter-finger modules. To the source of joint movement correlation, the factors from anatomic and physiological aspects are the main reasons [32]. From these two factors, our results can be well explained. The factors affect the correlation of finger joint movements including biomechanical connections between digits, functional organization of multitendoned finger muscles, and commands coming from central nervous system (CNS) [18, 32–33].

At first, for the biomechanical connections between the digits, the soft tissues of the web spaces couple adjacent fingers to some degree [34]. It is better known that the juncturae tendinium of extensor digitorum communis (EDC) produces the coupling movement in the adjacent fingers [35–36]. In addition, the flexor digitorum profundus (FDP) tendons are interconnected with the lumbrical muscles in the palm [36]. The interconnections between tendons of hand muscles and long tendons spanning finger joints will lead to the torque generation in adjacent joints [33]. Secondly, for the functional organization of multitendoned finger muscles, the extrinsic finger muscles (flexor digitorum superficialis, FDP, and EDC) have multiple tendons mapping to various joints of the hand, which lead to the mapping relation between joints and muscles is not a simple corresponding relation like one to one. The coupling movements of various joints will be occurred when each extrinsic finger muscles (flexor digitorum superficialis, FDP, and EDC) have a contraction [37–38]. Finally, for the commands coming from central nervous system (CNS), the influence to joint movement correlations can be divided into two parts. The first is that motoneuron pools innervating different finger muscles receive considerable shared central input [39–40]. Some of the investigations are based on the few specified actions designed by investigators, e.g. the independent movement of the specific finger [41–42]. However, to lots of grasp postures exploring the human hand functionality (like the HPD-RP built in this paper), the high use frequency of finger or joint means more together movements with other fingers or joints in different grasps. Therefore, as a command result of CNS, we think that the use frequency of different fingers or joints also has an indispensable influence to the finger or joint movement correlations.

Based on the aforementioned analysis and the study of this paper, for the inter-joint module correlations, compared with PIP and ABD joints, the MCP joints have a more complex mapping relation with muscles. The functional muscles of MCP joints are distributed not only in the forearm (extrinsic finger muscles, e.g. flexor digitorum superficialis, FDP, and EDC) but also the palm (intrinsic finger muscles, e.g. lumbrical muscles and palmar interossei muscles), and the contractions of these muscles will couple both the PIP joints and ABD joints to some degree. Moreover, the functional muscles distributed in the palm also have the extensive biomechanical connections with the actuated tendons of PIP and ABD joints. Therefore, the MCP joint module movement has the highest correlations with other joint modules. In contrast, to the functional muscles of wrist, they actuate the wrist joints independently with the hand joints, due to the less biomechanical connections between wrist and hand joints, and the perfect focal muscle-joint activation mapping relation [43]. Consequently, the correlation of the wrist module movement has the lowest correlations with other joint modules, across all MCP, PIP, ABD and wrist joint modules.

For the inter-finger module correlations, the hand digits except for thumb have the similar actuation structure in the anatomic and physiological aspects, thus we think that the use frequency of fingers across different grasp postures has a more practice impact on the move correlations for comparing the move correlations of these four fingers. The index finger is even used in all grasps, thus the index finger movement has the highest correlations compared with other finger modules. However, even though the thumb is very important in the grasp and used with a high frequency, it has an independent actuation structure in the anatomic and physiological aspects. Therefore, the thumb move more independently than other four fingers, the correlations of movement is low with other fingers. In this case, compared with the use frequency, we speculate that the anatomic and physiological structure of digits has a more important influence on the correlations of movement than the use frequency. At least, this is reasonable for the thumb.

### Postural synergies of human hand

There is a large dimensionality reduction in the number of DoFs of hand, which is also consistent with previous results. However, the results of information transmitted by PCs are lower than previous literatures. Compared with [19], the information transmitted by first two PCs decreases from >80% to 64%. This implies that changing relative position between human hand and objects has an effective influence to grasp postures. Moreover, the information transmitted by each subject PCs of hand postures (15DoFs) is very consistent (S2 Fig), which indicates that hand synergies are a general scheme.

For PCs of hand postures, compared with [14], [19] and [25], there are a greater proportion of movements in thumb joints especially the IP and ROT joint. This implies that the thumb plays a more important role in the tolerance grasping of different objects.

Furthermore, the highly-similar trends of the reconstructed angle error means the reconstructed angle error of hand posture is not sensitive to the changing relative position, which implies that changing relative position can’t cause the sudden increase of reconstructed angle error. Besides, the mean reconstructed angle errors are low (within 10°). These all shows the good performance for hand posture reconstruction in some applying areas (such as a design and control of the anthropomorphic hand).

### Postural synergies of human wrist

The wrist synergies are individually analyzed by PCA, which is little reported as far as we know. As the correlation between wrist module and joints of other modules is the lowest among different modules, the individual analysis of wrist synergies will be more practical. The result shows using two PCs to reconstruct the wrist posture can have a high information transmitted ratio among all ten subjects. Information transmitted by each subject PCs of wrist postures (3DoFs) is very similar, which indicates that wrist muscles show a synergy feature in people tolerance grasping. Result also shows the consistence of wrist PC among each subject is lower than that of the hand PC, because people can choose more kinds of wrist postures in tolerance grasping of different objects than that of hand postures.

Similar to the reconstructed angle error trends of hand postures, the reconstructed angle errors of wrist postures by first two PCs are also low (especially azimuth and roll) and highly-similar in different relative positions, which also shows the good performance for wrist posture reconstruction in some applying areas (such as a design and control of the anthropomorphic wrist). In contrast, the reconstructed angle errors of elevation are larger than that of azimuth and roll. This is likely due to the high height of cylinder, subjects can choose the upper or below area to accomplish the grasp more arbitrarily.

### For a design of anthropomorphic hand and wrist

As we all know, human hand is a template of anthropomorphic hand design. The high versatility and sophistication functionality of human hand makes the anthropomorphic hand design a systematic work, lots of indexes (such as grasp posture, force, speed, hand DoF configuration etc.) should be considered. The designer is confused how to design an anthropomorphic hand for a high performance. Under the inspiration, to the world’s most popular commercial prosthetic hands, we have a simple but more comprehensive evaluation of the anthropomorphic characteristics by a synthetic framework [44]. The results show the compliance, coupling speed ratio and DoF configuration is the lowest three indexes among 12 anthropomorphic indexes (whole configuration, size, weight, grasp speed, grasp force, compliance, length ratio, range of motion, rotation axis, coupling speed ratio, DoF configuration and grasp gesture). The compliance represents the flexible contact and compensation motions for adapting to the objects. Then, in terms of posture reconstruction, the compensation motions can be explained as small reconstructed angle error. In addition, the coupling speed ratio of joints is reflected in the implementation schemes to the reconstruction of human grasp posture. Furthermore, to the DoF configuration, the problem has to be solved is: how many DoF should be used to reconstruct the anthropomorphic grasp postures. Back to the research results of this paper, we try to utilize these experimental results for solving these problems.

Firstly, the reconstructed errors of joint angle can be set as the reference of compensation motions, which can inspire the designer that which fingers or joints should have a high compliance in mechanical design. From our results, mean reconstructed angle errors of hand different joints were mostly within 10° under the reconstruction of first four PCs. The great reconstructed angle errors were occurred in thumb IP joint, thumb MCP joint, index PIP joint, pinky MCP joint and pinky PIP joint, which suggests that thumb, index finger and pinky should be actuated more independently to compensate the angle errors. At first, because of the low correlation between thumb module and joints of other modules, thumb should be actuated individually apart with other four fingers. This individual actuation of thumb is a consistent conclusion with other studies, such as the high opposability of the thumb [45] [46], manipulation capabilities research [47] [48], experimental analysis [49] and anatomy research [43]. Then, for the pinky, an opposition with thumb is highlighted. The opposition helps to overcome the large relative position between hand and objects or large object size. In this case, one palm DoF below pinky is appropriate to improve the opposition performances, such as Shadow hand [50], Robonaut hand [51] and CEA hand [52].

Secondly, PC of hand postures is utilized to construct the coupling relationship among different joints for the reconstruction of the anthropomorphic grasp postures with a large reduction of active DoFs. An interesting use of this reconstruction knowledge is presented in [53] in which a 17 DoFs 5-fingered robot hand is successfully controlled with only two actuators. Following [53], some anthropomorphic hands are designed [28, 54–58].

Thirdly, information transmitted by PCs is utilized to solve the problem: how many active DoFs is needed to reconstruct human hand grasp postures. Based on our results, four active DoFs is suitable to reconstruct human hand grasp postures as low reconstructed angle errors (most < 10°) and high information transmitted ratio by PCs.

For the design of anthropomorphic wrist, the wrist should be designed individually based on lowest correlation between wrist and hand joints. This is consistent with the existing design of robotic arm [59–62]. Because of the high information transmitted ratio by PCs and low reconstructed angle errors, our results suggest that at least two active DoF are needed to design and control three DoF wrist by wrist synergies.

## Conclusions

This paper presented the hand and wrist postural synergies in tolerance grasping of various objects. The grasp postures collected from tolerance grasping of various objects could represent the grasp functionality of human hand more completely since the grasp tolerance, control principles and grasp adaptability (to different object sizes and shapes) are all considered. Based on the grasp postures collected from tolerance grasping of various objects, the correlation analysis among joints and modules newly defined was presented. The results indicate that coordination between wrist and hand joints is not obvious in tolerance grasping. In order to be applied more practically, the hand synergies and wrist synergies were analyzed separately. Moreover, the hand postures could be well reconstructed by first four PCs, as to wrist postures first two PCs were sufficient. Such findings are expected to facilitate the complete understanding of hand grasp and the design, control of the anthropomorphic hand and wrist.

## Supporting Information

S1 Data Human grasp posture data set in different relative positions. (XLSX)

S1 Fig CyberGlove calibration procedure. (TIF)

S2 Fig Information transmitted by PC of hand posture (15DoF) to each subject. (TIF)

S3 Fig First four PCs of hand posture to each subject and all ten subjects. (TIF)

S1 Table The calibration joints and actual joint angles in each calibration step. (DOCX)

## References

[1] Feix T, Bullock IM, Dollar AM. Analysis of human grasping behavior: Object characteristics and grasp type. IEEE Transactions on Haptics. 2014;7(3):311–323. 10.1109/TOH.2014.2326871 25248214

[2] Smeets JB, Brenner E. A new view on grasping. Mot Control. 1999;3:237–271. 10.1123/mcj.3.3.237 10409797

[3] Castiello U. The neuroscience of grasping. Nature Reviews Neuroscience. 2005; 6(9):726–736. 16100518 10.1038/nrn1744

[4] Jiang L, Liu Y, Yang D, Liu H. Analysis of Human Hand Posture Reconstruction Under Constraint and Non-constraint Wrist Position S Intelligent Robotics and Applications. Springer; 2015 p. 269–281.

[5] Bullock IM, Zheng JZ, Rosa S, Guertler C, Dollar AM. Grasp frequency and usage in daily household and machine shop tasks. IEEE Transactions on Haptics. 2013;6(3):296–308. 10.1109/TOH.2013.6 24808326

[6] Schlesinger IG. Der mechanische aufbau der künstlichen glieder Ersatzglieder und Arbeitshilfen. Springer; 1919 p. 321–661.

[7] Napier JR. The prehensile movements of the human hand. Journal of bone and joint surgery. 1956;38(4):902–913. 13376678 10.1302/0301-620X.38B4.902

[8] Kamakura N, Matsuo M, Ishii H, Mitsuboshi F, Miura Y. Patterns of static prehension in normal hands. American Journal of Occupational Therapy. 1980;34(7):437–445. 6446851 10.5014/ajot.34.7.437

[9] Cutkosky MR. On grasp choice, grasp models, and the design of hands for manufacturing tasks. IEEE Transactions on Robotics and Automation. 1989;5(3):269–279.

[10] Feix T, Pawlik R, Schmiedmayer H-B, Romero J, Kragic D, editors. A comprehensive grasp taxonomy. Robotics, Science and Systems: Workshop on Understanding the Human Hand for Advancing Robotic Manipulation; Seattle, WA, USA; 2009:2–3.

[11] Light CM, Chappell PH, Kyberd PJ. Establishing a standardized clinical assessment tool of pathologic and prosthetic hand function: normative data, reliability, and validity. Archives of physical medicine and rehabilitation. 2002;83(6):776–83. 12048655 10.1053/apmr.2002.32737

[12] Dalley S, Bennett D, Goldfarb M, editors. Functional assessment of the vanderbilt multigrasp myoelectric hand: A continuing case study. Proceedings of the IEEE International Conference on Engineering in Medicine and Biology Society. San Diego, California, USA; 2012: 4172–4175. 10.1109/EMBC.2014.6945044 PMC4476382 25571412

[13] Jeannerod M. Intersegmental coordination during reaching at natural visual objects. Attention and performance IX. 1981;9:153–168.

[14] Touvet F, Roby-Brami A, Maier MA, Eskiizmirliler S. Grasp: combined contribution of object properties and task constraints on hand and finger posture. Experimental brain research. 2014;232(10):3055–3067. 10.1007/s00221-014-3990-1 24888535

[15] Zheng JZ, De La Rosa S, Dollar AM, editors. An investigation of grasp type and frequency in daily household and machine shop tasks. Proceedings of the IEEE International Conference on Robotics and Automation. Shanghai, China; 2011:4169–4175

[16] Bullock IM, Feix T, Dollar AM, editors. Finding small, versatile sets of human grasps to span common objects. Proceedings of the IEEE International Conference on Robotics and Automation. Karlsruhe, Germany; 2013:1068–1075.

[17] Feix T, Bullock IM, Dollar AM. Analysis of human grasping behavior: Correlating tasks, objects and grasps. IEEE Transactions on Haptics. 2014;7(4):430–4041. 10.1109/TOH.2014.2326867 25532148

[18] Santello M, Baud-Bovy G, Jörntell H. Neural bases of hand synergies. Frontiers in computational neuroscience. 2013;7:1–15. 23579545 10.3389/fncom.2013.00023 PMC3619124

[19] Santello M, Flanders M, Soechting JF. Postural hand synergies for tool use. The Journal of Neuroscience. 1998;18(23):10105–10115. 9822764 10.1523/JNEUROSCI.18-23-10105.1998 PMC6793309

[20] Mason CR, Gomez JE, Ebner TJ. Hand synergies during reach-to-grasp. Journal of Neurophysiology. 2001;86(6):2896–2910. 11731546 10.1152/jn.2001.86.6.2896

[21] Grinyagin IV, Biryukova EV, Maier MA. Kinematic and dynamic synergies of human precision-grip movements. Journal of neurophysiology. 2005;94(4):2284–2294. 15917316 10.1152/jn.01310.2004

[22] Vinjamuri R, Mao ZH, Sclabassi R, Sun M, editors. Time-varying synergies in velocity profiles of finger joints of the hand during reach and grasp. Proceedings of the IEEE International Conference on Engineering in Medicine and Biology Society. Lyon, France; 2007: 4846–4849. 10.1109/IEMBS.2007.4353425 18003091

[23] Vinjamuri R, Sun M, Chang C-C, Lee H-N, Sclabassi RJ, Mao Z-H. Dimensionality reduction in control and coordination of the human hand. IEEE Transactions on Biomedical Engineering. 2010;57(2):284–295. 10.1109/TBME.2009.2032532 19789098

[24] Vinjamuri R, Sun M, Chang C-C, Lee H-N, Sclabassi RJ, Mao Z-H. Temporal postural synergies of the hand in rapid grasping tasks. IEEE Transactions on Information Technology in Biomedicine. 2010;14(4):986–994. 10.1109/TITB.2009.2038907 20071263

[25] Jarrassé N, Ribeiro AT, Sahbani A, Bachta W, Roby-Brami A. Analysis of hand synergies in healthy subjects during bimanual manipulation of various objects. J Neuroeng Rehabil. 2014;11:1–11. 25077840 10.1186/1743-0003-11-113 PMC4237861

[26] Huenerfauth M, Lu P. Calibration guide for cyberglove. The City University of New York, v4. 2009;4:22.

[27] Eckart C, Young G. The approximation of one matrix by another of lower rank. Psychometrika. 1936;1(3):211–8.

[28] Sun B, Xiong C, Chen W, Zhang Q, Mao L, Zhang Q, editors. A novel design method of anthropomorphic prosthetic hands for reproducing human hand grasping. Proceedings of the IEEE International Conference on Engineering in Medicine and Biology Society. Chicago, Illinois, USA; 2014:6215–6221. 10.1109/EMBC.2014.6945049 25571417

[29] Ma J, Thakor NV, Matsuno F. Hand and Wrist Movement Control of Myoelectric Prosthesis Based on Synergy. IEEE Transactions on Human-Machine Systems. 2015;45(1):74–83.

[30] Yang D, Gu Y, Liu R, Liu H. Dexterous motion recognition for myoelectric control of multifunctional transradial prostheses. Advanced Robotics. 2014;28(22):1533–1543.

[31] Pan L, Zhang D, Sheng X, Zhu X. Improving Myoelectric Control for Amputees through Transcranial Direct Current Stimulation. IEEE Transactions on Biomedical Engineering. 2015;62:1927–1936. 10.1109/TBME.2015.2407491 25730820

[32] Häger-Ross C, Schieber MH. Quantifying the independence of human finger movements: comparisons of digits, hands, and movement frequencies. The Journal of neuroscience, 2000 20(22): 8542–8550. 11069962 10.1523/JNEUROSCI.20-22-08542.2000 PMC6773164

[33] Schieber MH, Santello M. Hand function: peripheral and central constraints on performance. Journal of Applied Physiology, 2004 96(6): 2293–2300. 15133016 10.1152/japplphysiol.01063.2003

[34] von Schroeder HP, Botte MJ. The functional significance of the long extensors and juncturae tendinum in finger extension. The Journal of hand surgery, 1993 18(4): 641–647. 8349973 10.1016/0363-5023(93)90309-Q

[35] von Schroeder HP, Botte MJ, Gellman H. Anatomy of the juncturae tendinum of the hand. The Journal of hand surgery, 1990 15(4): 595–602. 2380523 10.1016/s0363-5023(09)90021-1

[36] Fahrer M. Interdependent and independent actions of the fingers The hand. Saunders, Philadelphia, 1981 399.

[37] Keen DA, Fuglevand AJ. Role of intertendinous connections in distribution of force in the human extensor digitorum muscle. Muscle & nerve, 2003 28(5): 614–622. 14571465 10.1002/mus.10481

[38] Keen D, Fuglevand A. Inter-tendonous connections play a minor role in the broad distribution of motor unit force in extensor digitorum. in Soc Neurosci Abstr. 1999.

[39] Bremner F., Baker J., and Stephens J., Correlation between the discharges of motor units recorded from the same and from different finger muscles in man. The Journal of Physiology, 1991 432: 355–380. 1886059 10.1113/jphysiol.1991.sp018389 PMC1181330

[40] Bremner F, Baker J, Stephens J. Variation in the degree of synchronization exhibited by motor units lying in different finger muscles in man. The Journal of physiology, 1991 432(1): p. 381–399. 1886060 10.1113/jphysiol.1991.sp018390 PMC1181331

[41] Kilbreath S, Gandevia S. Limited independent flexion of the thumb and fingers in human subjects. The Journal of Physiology, 1994 (Pt 3): 487–497. 7837104 10.1113/jphysiol.1994.sp020312 PMC1155766

[42] Reilly KT, Schieber MH. Incomplete functional subdivision of the human multitendoned finger muscle flexor digitorum profundus: an electromyographic study. Journal of neurophysiology, 2003 90(4): 2560–2570. 12815024 10.1152/jn.00287.2003

[43] Kapandji I. The Physiology of the Joints, vol. 3 Churchill Livingstone, Edinburg, Scotland 1974.

[44] Liu Y, Yang D, Jiang L, Liu H, editors. A synthetic framework for evaluating the anthropomorphic characteristics of prosthetic hands. Proceedings of the IEEE International Conference on Advanced Intelligent Mechatronics. Busan, Korea; 2015:877–884.

[45] Mouri T, Kawasaki H, Yoshikawa K, Takai J, Ito S, editors. Anthropomorphic robot hand: Gifu hand III. Proc. Int. Conf. ICCAS; Jeonbuk, Korea; 2002:1288–1293.

[46] Wang H, Fan S, Liu H, editors. An anthropomorphic design guideline for the thumb of the dexterous hand. Proceedings of the IEEE International Conference on Mechatronics and Automation. Chengdu, China; 2012:777–782.

[47] Chalon M, Grebenstein M, Wimböck T, Hirzinger G, editors. The thumb: Guidelines for a robotic design. Proceedings of the IEEE International Conference on Intelligent Robots and Systems. Taipei, Taiwan; 2010:5886–5893.

[48] Chalon M, Dietrich A, Grebenstein M. The Thumb of the Anthropomorphic Awiwi Hand: From Concept to Evaluation. International Journal of Humanoid Robotics. 2014;11(03):1450019.

[49] Saliba MA, Chetcuti A, Farrugia MJ. Towards the rationalization of anthropomorphic robot hand design: Extracting knowledge from constrained human manual dexterity testing. International Journal of Humanoid Robotics. 2013;10(02):1350001.

[50] Kochan A. Shadow delivers first hand. Industrial robot: an international journal. 2005;32(1):15–16.

[51] Lovchik C, Diftler MA, editors. The robonaut hand: A dexterous robot hand for space. Proceedings of the IEEE International Conference on Robotics and Automation. Michigan, USA; 1999:907–912.

[52] Martin J, Grossard M. Design of a fully modular and backdrivable dexterous hand. The International Journal of Robotics Research. 2014;33(5):783–798.

[53] Brown CY, Asada HH, editors. Inter-finger coordination and postural synergies in robot hands via mechanical implementation of principal components analysis. Proceedings of the IEEE International Conference on Intelligent Robots and Systems. San Diego, California, USA; 2007:2877–2882.

[54] Xu K, Liu H, Du Y, Zhu X. Design of an underactuated anthropomorphic hand with mechanically implemented postural synergies. Advanced Robotics. 2014;28(21):1459–1474.

[55] Li S, Sheng X, Liu H, Zhu X. Design of a myoelectric prosthetic hand implementing postural synergy mechanically. Industrial Robot: An International Journal. 2014;41(5):447–455.

[56] Rosmarin JB, Asada HH, editors. Synergistic design of a humanoid hand with hybrid dc motor-sma array actuators embedded in the palm. Proceedings of the IEEE International Conference on Robotics and Automation. Pasadena, California, USA; 2008:773–778.

[57] Chen W, Xiong C, Liu M, Mao L, editors. Characteristics analysis and mechanical implementation of human finger movements. Proceedings of the IEEE International Conference on Robotics and Automation. Hong Kong, China; 2014:403–408.

[58] Xu K, Liu H, Du Y, Sheng X, Zhu X, editors. Mechanical implementation of postural synergies using a simple continuum mechanism. Proceedings of the IEEE International Conference on Robotics and Automation. Hong Kong, China; 2014:1348–1353.

[59] Controzzi M, Cipriani C, Jehenne B, Donati M, Carrozza MC, editors. Bio-inspired mechanical design of a tendon-driven dexterous prosthetic hand. Proceedings of the IEEE International Conference on Engineering in Medicine and Biology Society. Buenos Aires, Argentina; 2010: 499–502. 10.1109/IEMBS.2010.5627148 21096539

[60] Zinck A, Stavdahl Ø, Biden E, Kyberd PJ. Design of a compact, reconfigurable, prosthetic wrist. Applied Bionics and Biomechanics. 2012;9(1):117–124.

[61] Takeda H, Tsujiuchi N, Koizumi T, Kan H, Hirano M, Nakamura Y, editors. Development of prosthetic arm with pneumatic prosthetic hand and tendon-driven wrist. Proceedings of the IEEE International Conference on Engineering in Medicine and Biology Society. Minnesota, USA; 2009:5048–5051. 10.1109/IEMBS.2009.5333668 19964378

[62] Bandara D, Gopura R, Hemapala K, Kiguchi K, editors. A multi-DoF anthropomorphic transradial prosthetic arm. Proceedings of the IEEE International Conference on Biomedical Robotics and Biomechatronics. São Paulo, Brazil; 2014:1039–1044.

