# Supplementary figures and images for: Analysis of Hand and Wrist Postural Synergies in Tolerance Grasping of Various Objects
Source: PLoS One. 2016 Aug 31;11(8):e0161772. doi: 10.1371/journal.pone.0161772 (PMC5007036; doi:10.1371/journal.pone.0161772)

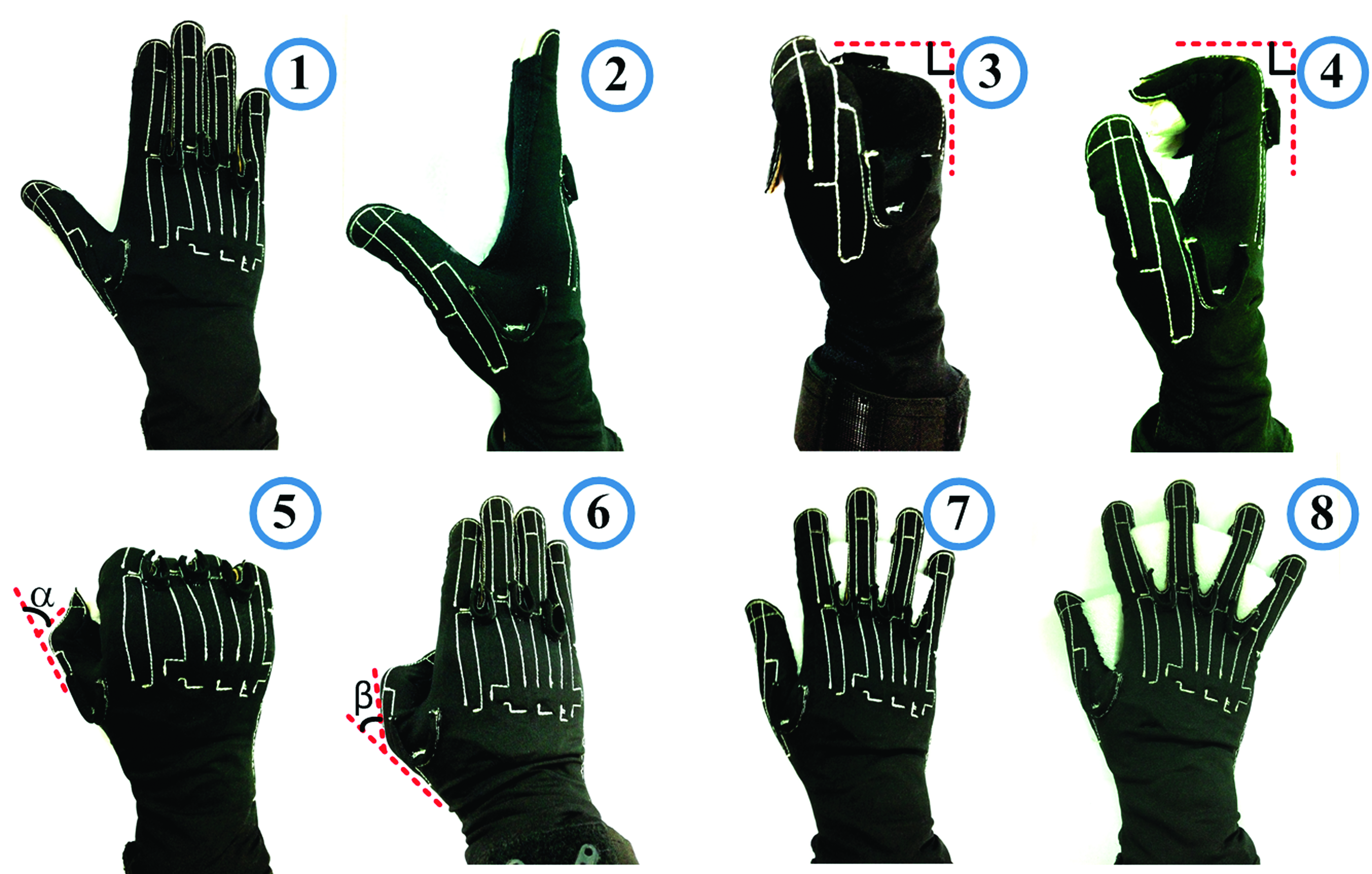

Supplement: S1 Fig — (TIF) [file pone.0161772.s002.tif]
