# Supplementary material for: Analysis of Hand and Wrist Postural Synergies in Tolerance Grasping of Various Objects
Source: PLoS One. 2016 Aug 31;11(8):e0161772. doi: 10.1371/journal.pone.0161772 (PMC5007036; doi:10.1371/journal.pone.0161772)
Supplement: S1 Table — (DOCX) [file pone.0161772.s005.docx]

**Table 1． The calibration joints and actual joint angles in each calibration step**

| **Step** | **Calibration joints** | **Actual joint angle** | **Step** | **Calibration joints** | **Actual joint angle** |
| --- | --- | --- | --- | --- | --- |
| **1** | PIP and MCP joints of four digits and thumb ROT joint | $0^{\circ}$ | **5** | thumb IP joint | $\alpha\text{=}10^{\circ},30^{\circ},50^{\circ}\text{，}70 ^{\circ}$ |
| **2** | thumb ROT joint | $90^{\circ}$ | **6** | thumb MCP joint | $\beta\text{=}10^{\circ},30^{\circ},45^{\circ}\text{,}60 ^{\circ}$ |
|  | thumb MCP joint | $0^{\circ}$ |  |  |  |
| **3** | MCP joints of four digits | $90^{\circ}$ | **7** | all ABD joints between adjacent fingers | $15^{\circ}$ |
| **4** | PIP joints of four digits | $90^{\circ}$ | **8** | all ABD joints between adjacent fingers | $30^{\circ}$ |
